# Street View Image-Based Road Marking Inspection System Using Computer Vision and Deep Learning Techniques †

**DOI:** 10.3390/s24237724

**Published:** 2024-12-03

**Authors:** Junjie Wu, Wen Liu, Yoshihisa Maruyama

**Affiliations:** 1 Nippon Koei Co., Ltd., 5-4 Kojimachi, Chiyoda-ku, Tokyo 102-8539, Japan; 2 Graduate School of Engineering, Chiba University, Inage-ku, Chiba 263-8522, Japan

**Keywords:** road markings, damage detection, computer vision, deep learning

## Abstract

Road markings are vital to the infrastructure of roads, conveying extensive guidance and information to drivers and autonomous vehicles. However, road markings will inevitably wear out over time and impact traffic safety. At the same time, the inspection and maintenance of road markings is an enormous burden on human and economic resources. Considering this, we propose a road marking inspection system using computer vision and deep learning techniques with the aid of street view images captured by a regular digital camera mounted on a vehicle. The damage ratio of road markings was measured according to both the undamaged region and region of road markings using semantic segmentation, inverse perspective mapping, and image thresholding approaches. Furthermore, a road marking damage detector that uses the YOLOv11x model was developed based on the damage ratio of road markings. Finally, the mean average precision achieves 73.5%, showing that the proposed system successfully automates the inspection process for road markings. In addition, we introduce the Road Marking Damage Detection Dataset (RMDDD), which has been made publicly available to facilitate further research in this area.

## 1. Introduction

In recent years, there has been rapid progress in the development of autonomous vehicles (AVs). The Society of Automotive Engineers (SAE) defines six levels of driving automation ranging from level 0 (no automation) to level 5 (full automation) [1]. Although there are no SAE level 5 AVs on the roads, some systems can support drivers away from the wheel, achieving SAE level 3 (conditional automation) capabilities. With the development of AV technologies, several aspects must be enhanced to embrace the era of AVs, including traffic management, road infrastructure adaptation, revenue and budgeting, liability and insurance, police and emergency services, and social justice and equity [2]. Road infrastructure adaptation is currently considered the most urgent problem to be solved. On the one hand, roads and other road infrastructure (e.g., lanes, markings, and signals) require adjustments and maintenance to accommodate AVs. However, implementing smart road technologies that are compatible with AVs will enhance road safety and efficiency.

The concept of road infrastructure adaptation for AVs should not only consider what new infrastructure needs to be established but also provide information about the support of road infrastructure for AVs. Thus, corresponding adjustments and maintenance can be performed to cope with the requirements of AVs. Before AVs came into play, there was already an established framework of road infrastructure adaptation called road classification, which was designed for conventional motorized vehicles, i.e., human drivers. Traditional road classification systems are called functional classification systems (FCSs) [3]. In previous decades, FCSs have been used by transportation planners to convey the level of service of roads. In recent years, rapid developments in the field of AVs have led to a renewed interest in road classification. Smart road classification (SRC) is widespread and primarily focuses on the degree of adaptation of AVs and connected vehicles [4]. A five-level SRC was established by the World Road Association, as shown in Table 1 [5]. Two indicators were considered to determine the SRC levels. These are the level of service for automated driving (LOSAD) and infrastructure support for automated driving (ISAD). The LOSAD comprises five levels that represent the readiness of a road for automated vehicles and the ISAD consists of five levels that focus on connectivity support. The SRC can help all stakeholders (road administration, road operators, equipment manufacturers, mobile network operators, users, and information management providers) know what to provide and expect from road facilities [6,7].

In the context of the SRC, a classification of the readiness of European highways to adopt connected, automated, and electric vehicles was proposed by the European Asphalt Pavement Association (EAPA) more recently, which is also known as the EAPA proposal. It should be noted that the EAPA is dedicated to asphalt paving for road construction and maintenance. This proposal defines six levels (from A to F) of roads to provide road information about the support level for AVs. One of the key parameters of the EAPA proposal is the condition of road markings. Four types of values of the condition of road markings—inadequate, adequate, satisfactory, and excellent—are used as important indicators to determine the EAPA proposal [8].

Road markings play a vital role in both AVs and SRC fields. A future road network will be a mix of human-driven vehicles and AVs, making road markings an important infrastructure element today and in the future [9]. Properly installed and well-maintained road markings provide abundant guidance and information to drivers and AVs. In contrast, damaged road markings pose significant challenges to human drivers and camera-sensor-based AVs because traffic safety is dependent on the visibility of road markings. Road markings inevitably wear out over time and thus the inspection and maintenance of road markings are crucial. However, the previous practice was to manually inspect the degree of road marking damage, which makes inspection and maintenance costly in terms of human and economic resources. To address these challenges, automated inspection systems using computer vision and deep learning techniques have gained attention. While several studies have focused on road damage detection, methods for detecting road marking damage have rarely been explored. One of the obstacles is the lack of publicly available datasets that specifically address road marking damage with detailed annotations and classifications.

In this study, we propose an automated road marking inspection system that leverages computer vision and deep learning techniques using street view images captured by a regular digital camera mounted on a vehicle. Our system includes a road marking damage assessment process that employs semantic segmentation, inverse perspective mapping (IPM), and image thresholding techniques to quantify the damage of road markings. Based on the assessment results, we develop road marking damage detectors using state-of-the-art object detection models, such as YOLOv11. Experimental results demonstrate that our system effectively automates the inspection process for road markings, achieving a mean average precision (mAP) of 73.5%. Another notable contribution of our work is the establishment of the Road Marking Damage Detection Dataset (RMDDD), which includes both original and augmented versions with detailed annotations of various types of road markings and their damage grades. The dataset can be downloaded at https://drive.google.com/file/d/1wYLujqrHKY0mOWHlxl0ftMhzerHKgl97/view?usp=drive_link (accessed on 25 November 2024). By making the RMDDD publicly available, we aim to facilitate further research in this area and enable other researchers to replicate and extend our work.

The remainder of this paper is organized as follows. Section 2 describes related work. Section 3 introduces the proposed method for inspecting road marking damage. The road marking damage detection dataset and results of the experiments are presented in Section 4. Section 5 presents our discussion and, finally, Section 6 concludes the paper.

## 2. Related Research

Various studies [10,11,12,13,14,15] that take advantage of the latest developments in deep learning and computer vision techniques have been conducted to improve the efficiency of road maintenance. Most of these studies have focused on road damage inspections. Among the best-known of these studies was the proposal of a computer vision and data-driven method-based solution to detect distress on road surfaces [10]. In a previous study, Chun et al. [11] developed an automated asphalt pavement crack detection method using an image-processing technique and a naive Bayes-based machine learning approach. Recently, the accurate real-time detection of road damage has become possible due to the development of several object detection frameworks, such as Faster R-CNN [16], YOLO series [17,18,19], and SSD [20]. For example, Maeda et al. [12,13] developed road damage detection methods using SSD Inception V2 and SSD MobileNet. In addition, the dataset published by these authors, named RDD-2018, in which eight different defects of the Japanese road network were proposed, has gained wide attention from researchers in this field.

Although road damage detection approaches have achieved convincing results, methods for detecting road marking damage have rarely been studied, with the exception of [21,22,23,24]. Vokhidov et al. [21] proposed a CNN-based method to recognize damaged arrow markings. Chong et al. [22] designed a hierarchical semantic segmentation strategy using U-Net to estimate the damage ratio of road markings based on damaged parts and regions. Unlike the semantic segmentation approach, Iparraguirre et al. built three types of road marking damage detectors using three different object detection architectures (Faster R-CNN, SDD, and EfficientDet) [23]. It should be noted that a new dataset of road marking defects is proposed in this study which is considered high complexity compared with the previous dataset. Kong et al. detected and assessed road marking defects at the city scale using a deep learning approach to contribute to road marking repair operations [24]. Table 2 summarizes representative studies in the fields of road damage detection and road marking damage detection.

## 3. Materials and Methods

This section presents the damage assessment process for road markings, followed by the inspection system proposed in this study. Specifically, the initial step involves the assessment of road marking damage, where the damage ratios of road markings are meticulously measured. Secondly, the RMDDD are systematically designed based on the outcomes of the previous step. In the final stage, road marking damage detectors are trained using the YOLO series models.

### 3.1. Road Marking Damage Assessment Process

**Figure 1:**
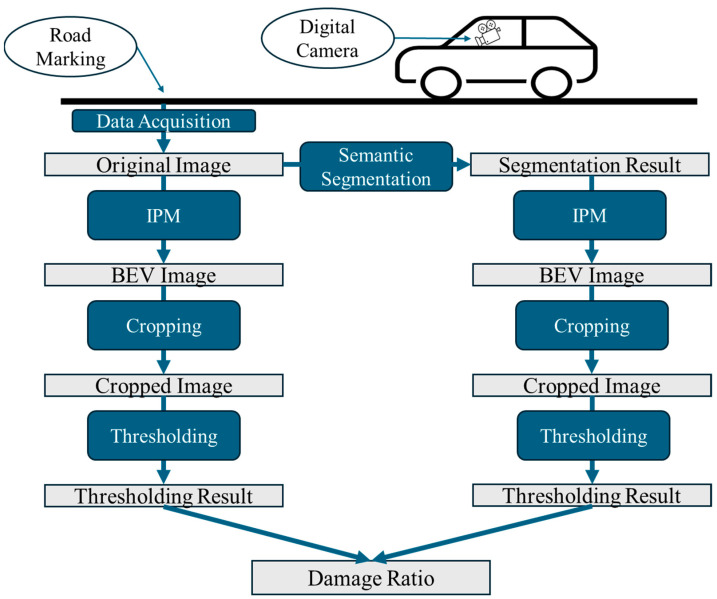
Overall flowchart of road marking damage assessment.

The fundamental idea of a road marking damage assessment is to measure the damage ratio of road markings based on their undamaged parts and the entire region [24]. Figure 1 shows the overall flowchart of the process. The process encompasses several steps, including data acquisition, semantic segmentation, inverse perspective mapping, image cropping, image thresholding, and the calculation of damage ratios. Each of these steps is explained in detail subsequently.

#### 3.1.1. Data Acquisition

**Figure 2:**
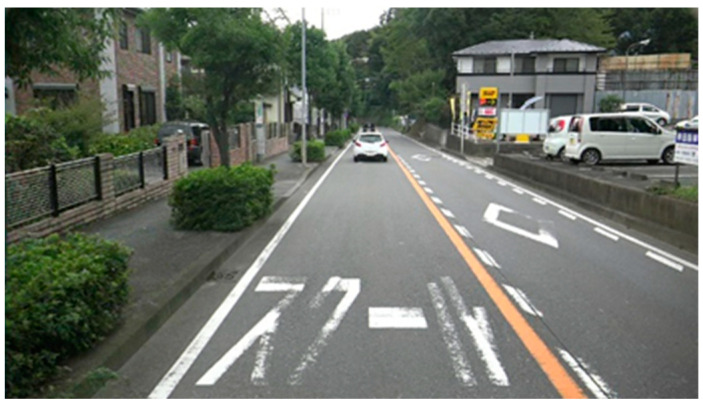
Example of street view image.

For data acquisition, a front-view camera mounted on a vehicle was used, as shown in Figure 1. The camera used in this study is a low-cost regular digital camera, making our proposed method cost-effective. Street view images were collected from three cities in Japan—Yokohama, Chofu, and Nogata—in November 2015, November 2015, and March 2017, respectively. Figure 2 shows an example of a captured street view image, and the road marking on the left side indicates a school zone.

#### 3.1.2. Semantic Segmentation

The second step is the semantic segmentation of the original image. In this study, the damage ratios of road markings were measured according to their undamaged parts and regions. Semantic segmentation was used to extract the integral regions of the road markings. It should be noted that the regions of road markings need to be completely extracted without defects, as this is crucial for the accurate calculation of the damage ratios. On the other hand, the undamaged part can be extracted from the original image using an image thresholding approach.

**Figure 3:**
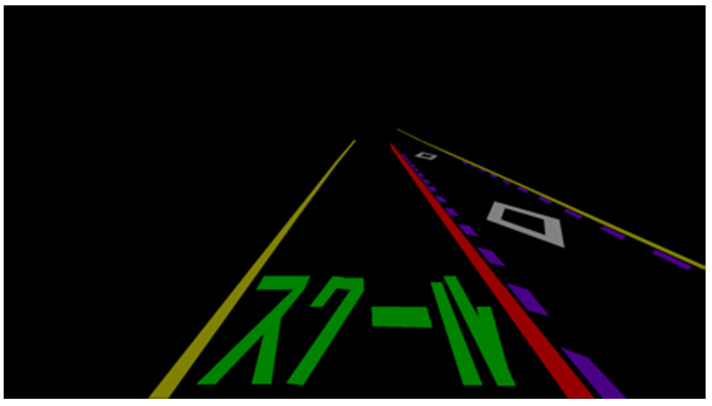
Example of segmentation result.

Since the problem of multiple-scale objects is prevalent in street view images [25], a neural network specifically designed for road marking segmentation known as a multiscale attention-based dilated convolutional neural network is used to handle the original image. This neural network uses multiple-scale images that are resized from the original image as inputs to learn the attention weights of each scale. Then, it merges the semantic predictions from each separate network to obtain the final output. Multi-scale inputs are used to improve segmentation accuracy by combining the advantages of each input scale. In addition, dilated convolution is adopted in the feature extraction process to utilize a larger range of spatial context information [25]. The performance analysis shows that the method outperforms other state-of-the-art models by addressing the problem of multiple-scale objects in street view images. In addition, the ablation experiments show that the neural network yields the best results by combining multiscale attention and dilated convolution. The segmentation result is promising because the neural network successfully extracts the entire road marking region. Figure 3 shows an example of the segmentation result shown in Figure 2.

#### 3.1.3. Inverse Perspective Mapping

**Figure 4:**
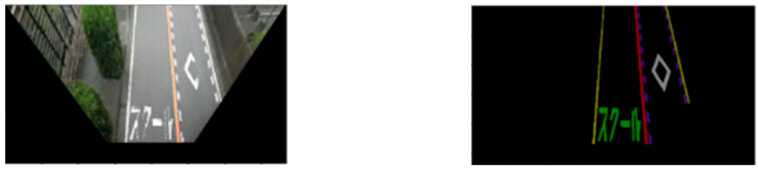
Examples of the obtained BEV images (**left**: original image; **right**: segmentation result).

In the captured street view images, the pixels occupied by each road marking are different. As shown in Figure 2, the distant road marking of the approach to the pedestrian and bicycle crossings (prismatic road marking on the right side of the road) is thinner than the nearby marking. This is not conducive to a damage assessment of the road markings [24]. Moreover, the street view image is horizontal, and the perspective effect can negatively affect the road marking damage assessment. Therefore, IPM is adopted to convert the street view images into a bird’s eye view (BEV) images. Generally, IPM maps the pixels of an image from a horizontal to a vertical view through a homography matrix, which is a transformation matrix. In this study, the Open Source Computer Vision Library (OpenCV) [26], which provides more than 2500 optimized algorithms for computer vision tasks, was used to conduct the IPM on both the original image and the segmentation result. We primarily used the functions of cv2.getPerspectiveTransform, which takes the four pairs of corresponding points as input and outputs the transformation matrix, and cv2.warpPerspective, which applies the transformation matrix to the input image to obtain the BEV image. Figure 4 shows the BEV images obtained in this study through the IPM process.

#### 3.1.4. Image Cropping

**Figure 5:**
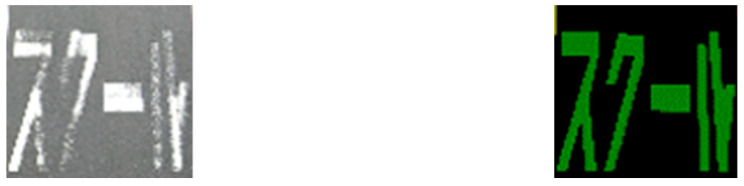
Examples of the cropped road marking images (**left**: original image; **right**: segmentation result).

To conduct an independent damage assessment of road markings, each instance must be cropped from both the original image and the segmentation result. Figure 5 shows examples of cropped road marking images.

#### 3.1.5. Image Thresholding

**Figure 6:**
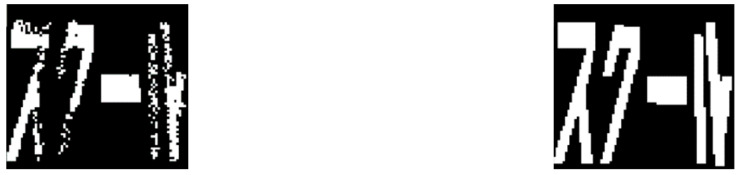
Examples of the thresholding results (**left**: original image; **right**: segmentation result).

Because the goal of the assessment process is to measure the damage ratios of road markings according to their areas, it is necessary to convert the areas of the undamaged parts and regions to the number of pixels in the image for calculation. Image thresholding can perform the basic segmentation of an image and convert it into a binary image, where the pixels are either 0 or 1 (or 255) [27]. Hence, image thresholding is employed to extract the undamaged parts and regions of road markings from the cropped images obtained in the previous step. Figure 6 shows the thresholding results obtained using OpenCV.

#### 3.1.6. Damage Ratio

The damage ratio of road markings can be measured as follows:

(1) $$R=1-\frac{N_{ud}}{N_{r}}$$

where $N_{ud}$ is the number of pixels in the undamaged part of the road markings, $N_{r}$ is the number of pixels in the road marking region, and R represents the damage ratio of the road markings. Because the IPM process eliminates the perspective effect, every pixel in the binary image obtained in the previous step has the same area in reality. Thus, the ratio of the area of the undamaged part to that of the road marking region can be regarded as the ratio of the number of pixels in the undamaged part to the number of pixels in the road marking region. In Figure 6, the original image shows an undamaged area containing 2573 pixels, while the total number of pixels in the region indicated by the segmentation result is 3841, leading to a damage ratio of 0.33.

### 3.2. Road Marking Damage Inspection System

**Figure 7:**
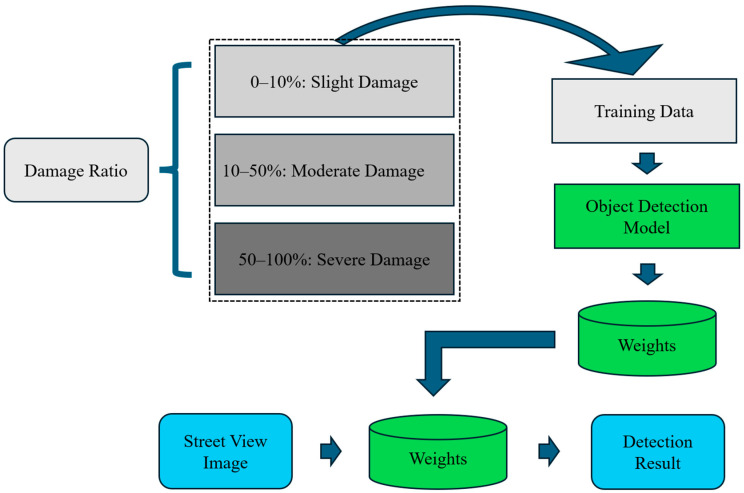
Flowchart of road marking damage inspection system.

A flowchart of the road marking damage inspection system using the object detection approach is shown in Figure 7. Inspired by [24], the damage ratios (*R*) of the road markings are divided into three grades: slight, moderate, and severe. A damage ratio of 0–10% is regarded as slight damage, which indicates that there is no need for maintenance. A damage ratio of 10–50% is regarded as moderate damage, which means that the defects must be repaired. A damage ratio of 50–100% is regarded as severe damage, which means that the defects require urgent responses. The results of the road marking damage assessment are then used as labeled data to train the object detection models. The object detection model can detect and locate the objects of interest in an image, which, in this study, are road markings with damage-grade labels. There are many excellent object detection algorithms, including Faster R-CNN [16], the YOLO series [17,18,19,28], and SSD [20]. After training and model validation, the obtained weights could automatically detect road markings with damage-grade labels from a street view image.

## 4. Results

### 4.1. RMDDD

Based on the road marking damage assessment process described in Section 3.1, we developed the RMDDD. This dataset is a significant outcome of our study and serves as a foundation for training and evaluating road marking damage detection models. For the road marking damage assessment, 1000 street view images with 3909 road marking instances were processed. Specifically, the damage ratios of road markings were measured during the road marking damage assessment process. As mentioned earlier, damaged road markings are divided into three grades according to their damage ratios: slight, moderate, and severe damage. On this basis, we divided road markings into line, arrow, block, and word and number markings according to their shapes. This means that the RMDDD contains 12 classes (see Table 3).

An annotation tool called Roboflow is used to manually annotate the street view images. Road markings are annotated with bounding boxes, which is a commonly used method of annotation in object detection. The original street view image consists of 1920 × 1080 pixels. Since the ratio of road markings to the whole image is not small, most of the objects are large, and thus the images are resized to 640 × 640. Large objects can be detected better using small-size images [25].

**Figure 8:**
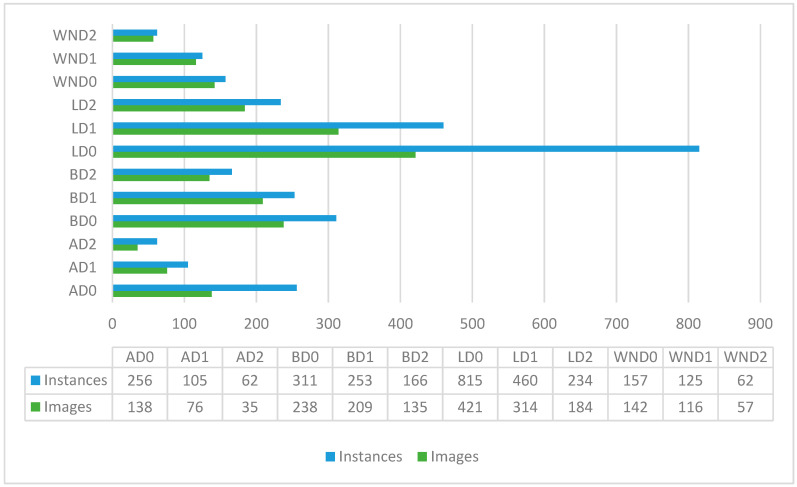
Number of instances and images in each class.

**Figure 9:**
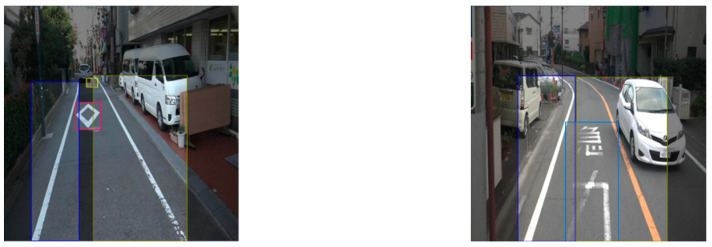
Examples of the RMDDD.

Given the limitations of the original RMDDD, which consists of only 1000 images and 3909 instances as mentioned before, data augmentation approaches are applied to increase the number of instances and images of each class. Despite these efforts, a data imbalance problem remains in the original RMDDD. As shown in Figure 8, the LD0 class is over-represented, whereas the classes AD1, AD2, BD2, WND0, WND1, and WND2 are under-represented. The imbalance in the number of instances and images across different classes could potentially impact the detector’s ability. To address this issue, data augmentation was specifically applied to the under-represented classes (AD1, AD2, BD2, WND0, WND1, and WND2). Horizontal and vertical flipping, cropping, and additive brightness adjustments were adopted to generate a synthetic dataset, referred to as the augmented RMDDD, which consists of 2640 images and 10,453 instances. The augmented RMDDD comprises 2440 training images, along with 100 validation images and 100 test images. Examples of the road marking damage detection datasets are shown in Figure 9.

### 4.2. Road Marking Damage Detector

**Figure 10:**
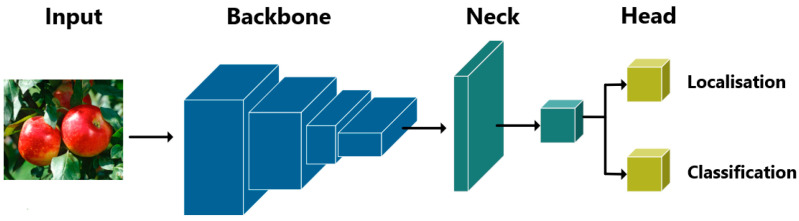
Abstract architecture of YOLO series models [32].

Detectors based on the YOLOv8 [19], YOLOv9 [29], YOLOv10 [30], and YOLOv11 [31] architectures have been built to address the proposed RMDDD. As we know, a significant advancement in the field of object detection was marked by the introduction of the YOLO algorithm, developed by Redmon et al. [17]. This algorithm revolutionized the approach to real-time object detection by enabling simultaneous detection and classification within a single neural network framework. As shown in Figure 10, the abstract architecture of the YOLO series is comprised of three essential components. Firstly, the backbone functions as the main feature extractor, employing convolutional neural networks to convert raw image data into multi-scale feature maps. Secondly, the neck serves as an intermediary processing layer, utilizing specialized structures to aggregate and refine feature representations across various scales. Lastly, the head acts as the prediction mechanism, producing the final outputs for object localization and classification based on the refined feature maps [28,32].

**Figure 11:**
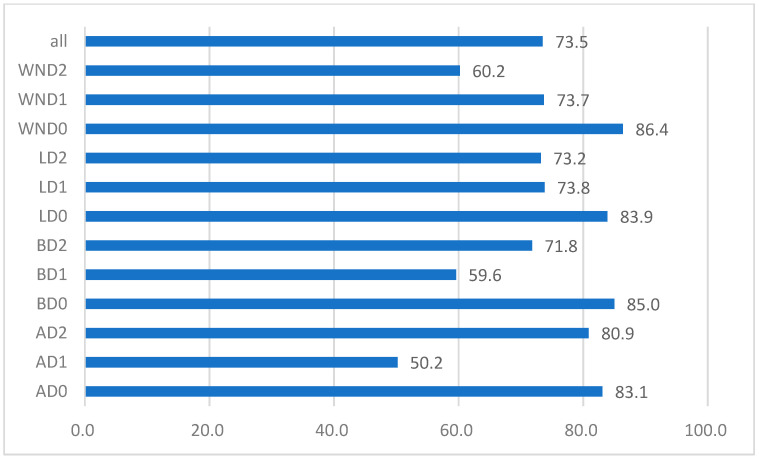
mAP values of each class obtained by the model of YOLOv11x.

The detectors are trained using an NVIDIA A100 GPU and PyTorch 2.4.1 is used for the deep-learning framework. After exploring several hyperparameters, the weights of the best fit are generated. The optimizer used is a stochastic gradient descent (SGD) with a momentum of 0.937. The learning rate and weight decay are set as 0.01 and 0.0005, respectively. The models are trained for 100 epochs, with a batch size of 16. The detailed hyper-parameters are listed in the configuration file, which is stored in the repository alongside the RMDDD. After the training, the models are evaluated using the test set. The mean average precision (mAP), precision, and recall are used as evaluation metrics for assessing the performance of the YOLO models on the RMDDD. The results are presented in Table 4. The YOLOv11x model demonstrates the best overall performance, achieving a mAP50 of 73.5%. It also shows strong results in precision and recall, with values of 67.3% and 72.4%, respectively. Additionally, the YOLOv11x model achieves an inference speed of 4.0 milliseconds per image, translating to approximately 250 frames per second, which is suitable for real-time applications. In comparison, the YOLOv11n model achieves a faster speed of 385 frames per second, making it an excellent choice for applications where speed is critical. Figure 11 shows the mAP values of each class obtained with YOLOv11x model. Overall, more than half of the classes have mAP values greater than 73%. A reasonably accurate baseline for road marking damage detection is made in this study.

**Figure 12:**
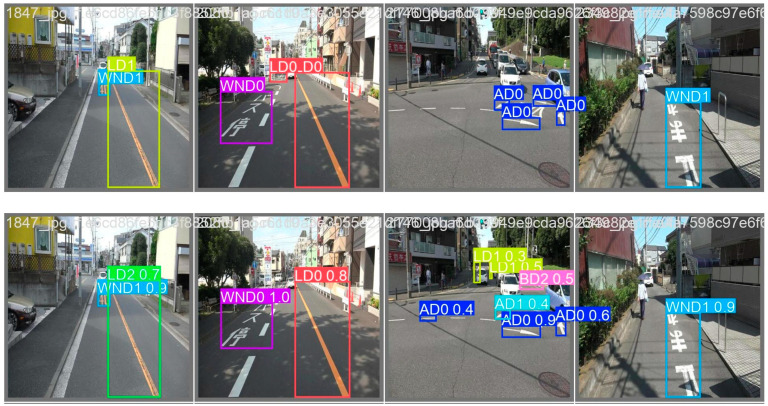
Visualization of road marking damage detection results of YOLOv11x on four test images (first row: ground truth; second row: results of prediction).

Figure 12 presents the visual results of road marking damage detection based on the YOLOv11x model on four test images. The first row corresponds to the ground truth labels, whereas the second row represents the predictions performed by the YOLOv11x-based detector. It can be seen that road marking damages are successfully detected by the bounding boxes.

## 5. Discussion

### 5.1. RMDDD

To the best of our knowledge, the dataset constructed by Iparraguirre et al. [23] represents the first effort to create a dataset specifically for road marking damage detection. However, only lane defects are annotated in their dataset. In contrast, the RMDDD proposed in this study is more extensive and detailed, encompassing a broader range of road markings. Firstly, the RMDDD proposed in this study covers road markings of different shapes, which are divided into four types: line, arrow, block, and word and number. Secondly, the damage ratios of the road markings are precisely calculated and classified into three grades: slight, moderate, and severe. Overall, the RMDDD is more detailed and comprehensive than the previous dataset.

**Figure 13:**
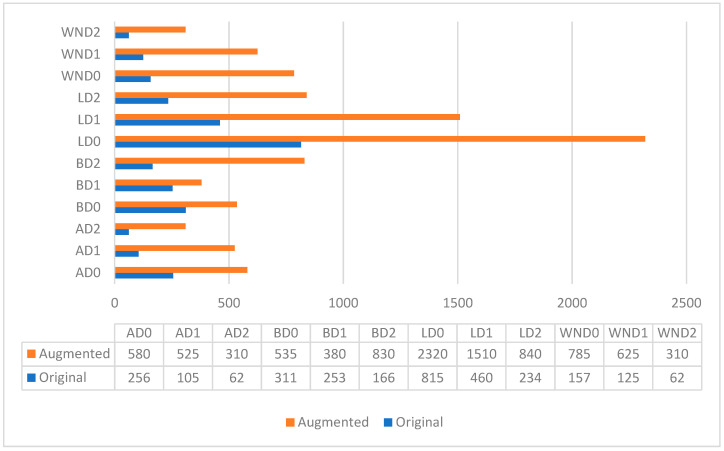
Number of instances in each class of the original and augmented RMDDD.

Moreover, data augmentation approaches are implemented to generate the augmented version. Figure 13 shows the number of instances in each class of both the original and augmented RMDDD. Compared to the original version, the data imbalance problem is improved in the augmented version by implementing data augmentation specifically for the under-represented classes. However, the data imbalance problem persists. This is primarily because the augmented instances are often derived from the same original images, leading to a proportional increase in the over-represented classes as well. Consequently, the relative imbalance between the over-represented (LD0) and under-represented classes (AD1, AD2, BD2, WND0, WND1, and WND2) remains. To address the issue of data imbalance, future research could explore advanced methods such as generative adversarial networks (GANs) [33] for synthetic data generation. GANs could be used to create additional instances for under-represented classes, potentially reducing bias and improving model performance. For example, Shrivastava et al. exploit GANs to enhance the realism of synthetic images. Their method not only refines the visual fidelity of generated images but also aligns them more closely with real-world data distributions, which significantly improves the training effectiveness of object detection models. This technique demonstrates a crucial application of GANs in augmenting training datasets, particularly when real annotated data is scarce or when certain classes are under-represented [34]. Similarly, Zhang et al. explore a novel approach where GANs are modified to generate synthetic instances of infrequent classes, thus addressing class imbalance directly. This method involves dynamically adjusting the generation process based on feedback from the object detection model’s performance, which helps ensure that the synthetic data is not only diverse but also tailored to improve the detector’s ability to recognize less frequent objects [35].

### 5.2. Road Marking Damage Detector

The road marking damage detectors are trained using pretrained models and then fine-tuned. Because road markings with three types of damage grades are unique, we also try to train them from scratch. The results show that a mAP of 69.3% is obtained when training YOLOv11x from scratch. This is significantly lower than the 73.5% mAP obtained using the pretrained model. This suggests that transfer learning is effective for the road marking damage detection task.

**Figure 14:**
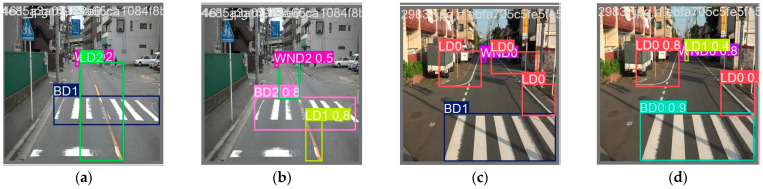
Samples of test images with labels (**a**,**c**) and predictions of incorrect detection (**b**,**d**).

The mAP of road markings with moderate damage is lower than that of the others. Because the damage ratio of road markings with moderate damage is defined as 10–50%, road markings with a damage ratio slightly greater than 10% are incorrectly detected as having slight damage. In addition, road markings with a damage ratio slightly lower than 50% are incorrectly detected as severely damaged. This can be observed in the visual analysis shown in Figure 14. BD1 in the test image is incorrectly detected as BD2 (see Figure 14a,b). BD1 in another test image is incorrectly detected as BD0 (see Figure 14c,d).

## 6. Conclusions

This study proposes an automatic road marking inspection system using street view images. Properly installed and well-maintained road markings provide abundant guidance and information for drivers and AVs. However, few studies have addressed the detection of road marking defects. To address this issue, a road marking damage assessment process that employs semantic segmentation, IPM, and image thresholding techniques is proposed. The core idea is to compare the areas of the undamaged part of the road markings with their damaged regions. This approach results in a significant contribution of this study, which is the creation and public release of the RMDDD. The augmented version of the RMDDD contains 2640 images with 10,453 instances of road markings, categorized into four types: line, arrow, block, and word and number. The damage for each road marking is further classified into three grades: slight, moderate, and severe. By making the RMDDD publicly available, we aim to facilitate further research in this area and enable other researchers to replicate and improve upon our results. State-of-the-art models, specifically the YOLO series, are employed to train road marking damage detectors. Experimental results indicate that the detector utilizing the YOLOv11x model achieves a promising performance of 73.5% mAP, automatically and effectively detecting damage to road markings. This system represents a significant advancement in the field of road marking inspection, offering a robust solution for maintaining road safety and efficiency. Future research will focus on deploying the proposed system in real-world scenarios. This will help to validate the effectiveness and adaptability of this system outside of controlled environments, ensuring reliability under various conditions.

The road marking damage assessment process should be improved, particularly in terms of the image cropping process. In this study, the image cropping process is conducted manually; however, this should be replaced by an automated process to eliminate human error. Training a detector to recognize road markings based on their geometric shapes is an effective method to automate image cropping and reduce human errors, enhancing the precision of the RMDDD. Furthermore, future efforts should focus on exploring advanced methods, such as synthetic data generation, to continuously improve the balance and quality of road marking damage detection datasets.

## Figures and Tables

**Table 1:** The content of smart road classification.

Type of Road Segments	Content
Human-way (HU)	Do not support automation.
Assisted-way (AS)	Present partial support for automation, with remarkably fewer disengagements than on HU road segments.
Automated-way (AT)	Present similar physical characteristics to AS road segments, but also present connectivity capabilities that could help connected vehicles prevent and avoid disengagements.
Full Automated-way (FA)	Present full support for SAE level 4 vehicles and good connectivity capabilities.
Autonomous-way (AU)	Present full support for SAE level 4 vehicles and exceptional connectivity capabilities. They can only be used by SAE level 4 and 5 vehicles.

**Table 2:** Representative studies in the field of road damage detection and road marking damage detection.

Road Damage Detection			Road Marking Damage Detection		
Reference	Approach	Year	Reference	Approach	Year
[10]	Image classification	2014	[21]	Image classification	2016
[11]	Image segmentation	2015	[22]	Image segmentation	2021
[12]	Object detection	2018	[23]	Object detection	2022
[13]	Object detection	2020	[24]	Image segmentation	2022

**Table 3:** Classification system of the proposed RMDDD.

Shape	Grade of Damage	Class Name
Arrow	Slight damage	AD0
	Moderate damage	AD1
	Severe damage	AD2
Line	Slight damage	LD0
	Moderate damage	LD1
	Severe damage	LD2
Block	Slight damage	BD0
	Moderate damage	BD1
	Severe damage	BD2
Word and Number	Slight damage	WND0
	Moderate damage	WND1
	Severe damage	WND2

**Table 4:** Performance statistics of the YOLO models on the RMDDD.

Model	mAP50 (%)	mAP50-95 (%)	Precision (%)	Recall (%)	Speed (ms per Image)	Layers	GFLOPs
YOLOv8x	72.4	51.4	68.8	68.5	3.9	268	257.4
YOLOv9-e	73.1	53.1	65.9	75.8	5.3	467	102.5
YOLOv10x	68.7	48.6	61.0	68.7	6.1	503	169.9
YOLOv11x	73.5	53.3	67.3	72.4	4.0	464	194.5
YOLOv11n	66.8	46.9	63.3	65.7	2.6	238	6.3

## Data Availability

The dataset can be downloaded at https://drive.google.com/file/d/1wYLujqrHKY0mOWHlxl0ftMhzerHKgl97/view?usp=drive_link (accessed on 25 November 2024).
